# Giant Idiopathic Angioid Streaks

**DOI:** 10.18502/jovr.v15i2.6742

**Published:** 2020-04-06

**Authors:** Brijesh Takkar, Anubha Rathi, Pradeep Venkatesh, Atul Kumar

**Affiliations:** ^1^ Department of Ophthalmology, All India Institute of Medical Sciences, Bhopal, India; ^2^ Smt Kanuri Santhamma Centre for Vitreoretinal Diseases, LV Prasad Eye Institute, Hyderabad, India; ^3^ Department of Ophthalmology, Dayanand Medical College, Ludhiana, India; ^4^ Retina and Uvea Services, Dr R P Centre for Ophthalmic Sciences, All India Institute of Medical Sciences, New Delhi, India

**Keywords:** Angioid Streak, Choroidal Neovascular Membrane, Pseudoxanthoma Elasticum, Swept Source Optical Coherence Tomography

## Abstract

**Purpose:**

To present a case of gigantic idiopathic angioid streaks.

**Case report:**

A young male presented with macular choroidal neovascular membrane (CNVM) and peripheral retinal hemorrhages secondary to angioid streaks. Swept source optical coherence tomography (SSOCT) and ultrawide field imaging were performed. The latter revealed extension of the angioid streaks up to the equator in both eyes. SSOCT showed breaks in the retinal pigment epithelium-Bruch's membrane complex in the area of peripheral retinal hemorrhages. The patient was extensively worked up for systemic associations, and the only significant finding was a long history of steroid abuse in the past.

**Conclusion:**

Advanced imaging techniques helped to diagnose angioid streaks in this patient. The possible role of steroid abuse in accentuating the presentation of angioid streaks may be explored further.

##  INTRODUCTION

Angioid streaks (AS) are dehiscences in the Bruch's membrane, clinically seen to radiate from the peripapillary area and accompanied by atrophy of the overlying retinal pigment epithelium (RPE). Because of the blood vessel-like appearance, they were earlier believed to have a vascular origin but later proved to be cracks in the Bruch's membrane with secondary calcification. They may or may not have systemic association, the most common being pseudoxanthoma elasticum.^[1,2]^ The streaks are usually restricted to the posterior pole and very rarely involve the retinal periphery.^[2]^

In this imaging report, we present ultrawide field (UWF) and swept source optical coherence tomography (SSOCT) imaging features of a case of idiopathic AS that extended from the optic disc to the pre-equatorial region. Possible role of steroid abuse toward the unusual presentation in this patient is also discussed.

##  CASE REPORT

All procedures performed in the present study was approved by the All India Institute of Medical Sciences, New Delhi Research Committee.

**Figure 1:**
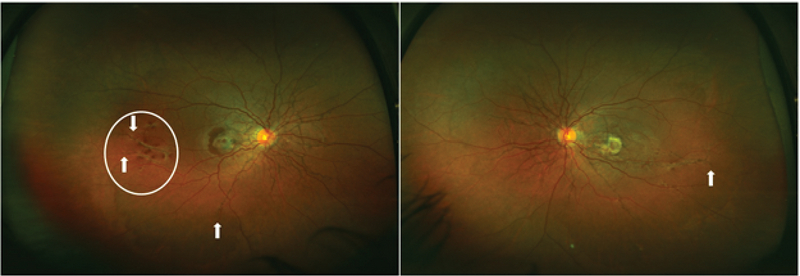
Ultrawide field fundus images of the right and left eyes showing a peripapillary meshwork of angioid streaks at the posterior pole of both eyes along with peripheral extension up to the pre-equatorial region (arrow). A CNVM is noticeable in the right macula along with subretinal blood, while the left eye has a dense scar in the macular region. Additionally, right eye has an area with peripheral retinal hemorrhages bounded by two peripheral streaks (encircled).

A 35-year-old male presented with low vision in right eye for two months that was painless and gradual in onset. He had a history of being treated with two intravitreal injections of bevacizumab for subfoveal choroidal neovascular membrane (CNVM) in the left eye one year back. The best corrected visual acuity was 6/36 in the right eye and 6/60 in the left eye. Anterior segment examination and intraocular pressure were within normal limits in both eyes. Fundus examination revealed a spider web-like network of blackish–brown colored radially oriented interconnected streaks that emanated from the peripapillary region in both eyes. These streaks were seen to reach up to the pre-equatorial region of the inferior and temporal quadrants of both eyes on UWF imaging [Figure 1] (Optos, Optos plc, Dunfermline, UK). Further, a subfoveal CNVM with subretinal bleed was seen in the right eye, while a disciform scar was noticed in the macular region of the left eye. Extramacular retinal hemorrhages were visualized in temporal immediate periphery of both eyes [Figure 1]. The patient was clinically diagnosed to have AS in both eyes, with active CNVM in the right eye and macular scar in the left eye.

**Figure 2:**
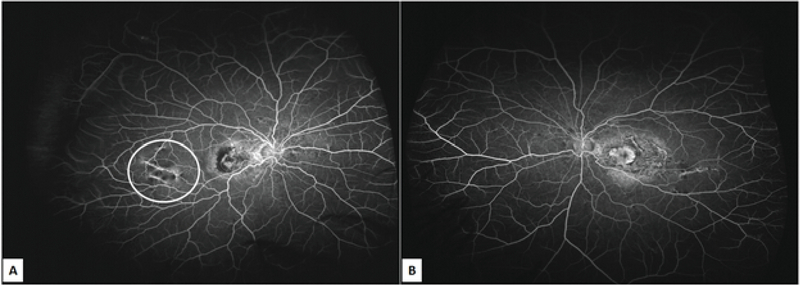
Ultrawide field fluorescein angiogram of the patient showing (A) CNVM in the macular region of right eye and (B) scarring in the macula of left eye. Encircled area shows minimal dye leakage corresponding to the peripheral angioid streaks that resulted in bleeding.

**Figure 3:**
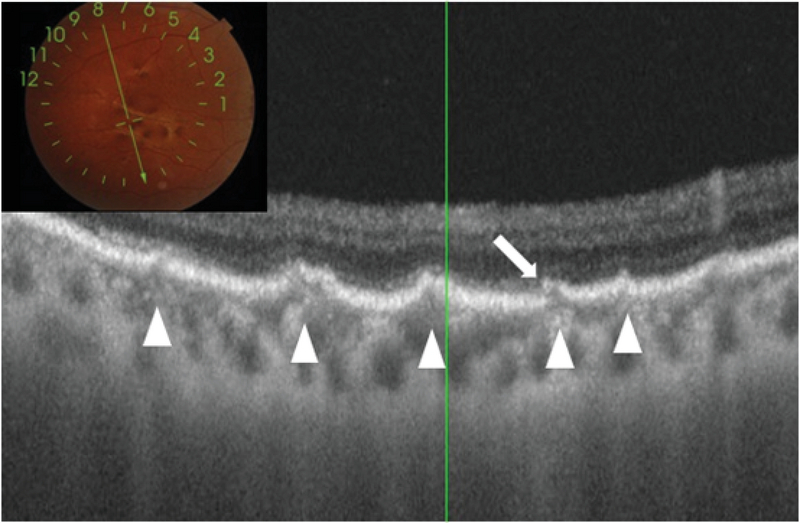
Swept source optical coherence tomography through the temporal peripheral area of the right eye having hemorrhages. The scan has been acquired through an area of hemorrhage between two peripheral angioid streaks (inset). Undulations can be seen in multiple areas at the level of the RPE (arrowheads). One of these areas also shows a break in the epithelium (arrow).

**Figure 4:**
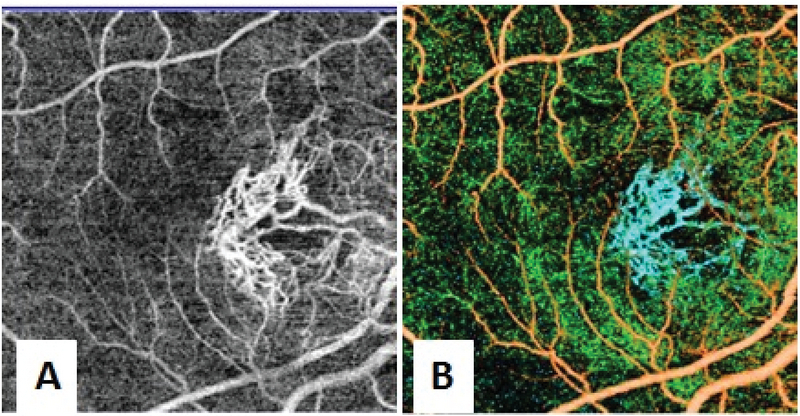
Swept source optical coherence tomography angiography of the macula of the right eye scanned over an area of 4.5 mm × 4.5 mm. (A) The imaging slab has been at the level of the choriocapillaris. A well-defined meshwork of mature vessels of the CNVM can be seen along with the projection artifact of the retinal vessels. (B) In the figure showing the composite angiogram, CNVM is seen as blue colored vessels.

UWF fluorescein angiography of the right eye revealed an actively leaking subfoveal CNVM with hypofluorescence corresponding to hemorrhages, while the left macula showed only staining [Figure 2]. The AS were visualized as curvilinear lines with alternating areas of hypo and hyperfluorescence in both eyes. Additionally, the temporal AS of the right eye that passed underneath the CNVM had minimal leakage in its peripheral, most extensions corresponding to the area of fresh retinal hemorrhages [Figure 2]. SSOCT imaging (Topcon DRI OCT Triton, Topcon, Tokyo, Japan) of the AS in this area showed multiple elevations corresponding to the AS along with breaks in the RPE-Bruch's membrane complex [Figure 3]. While the left eye had no subretinal fluid, SSOCT angiography of the right eye revealed a type 2 CNVM in the macular region, while no such membrane was seen in the area corresponding to peripheral retinal hemorrhages [Figure 4].

The patient was worked up for systemic associations of AS, and pseudoxanthoma elasticum, Paget's disease and anemia were ruled out.^[2,3]^ The patient, however, gave the history of long-term over-the-counter steroid abuse in the past with the intention of weight gain and increase in muscle mass. He had consumed varying doses of oral methylprednisolone ranging from 10 to 40 mg daily for a period of three to five years up to the current presentation. He also had a history of long-term use of anabolic steroids. The patient was counseled against further self-medication and was offered intravitreal anti-VEGF injection in the right eye. The patient however did not follow-up for the treatment despite adequate counseling.

**Figure 5:**
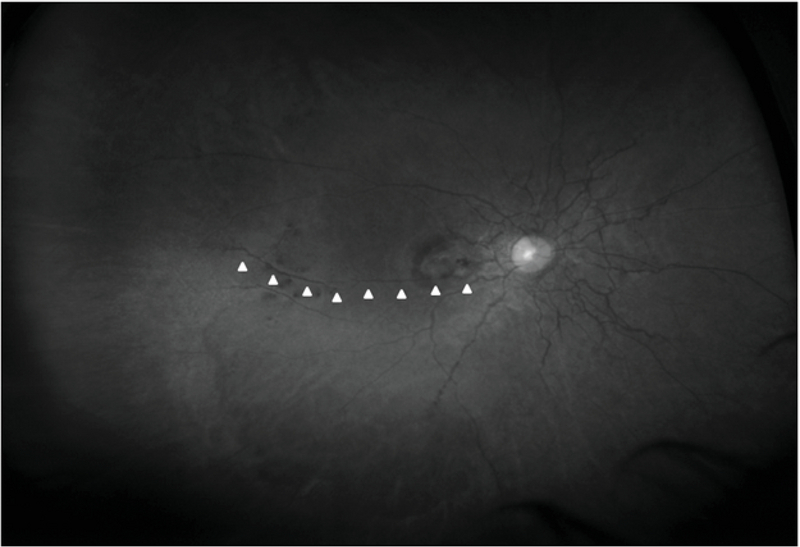
Ultrawide field red free fundus photograph of the right eye showing the peripheral angioid streak as an extension of the central complex.

##  DISCUSSION

Angioid streaks are typically restricted to the posterior pole, and very rarely extend to the peripheral areas. They are typically described to be a few mm in length from the optic disc, and mostly much less than half a mm in width.^[2]^ It is possible that the absence of UWF technology did not previously allow for clinical documentation of such large streaks. At the same time, it should be noted that studies have also been done on enucleated eyeballs with AS,^[2,4]^ a fact that makes UWF findings of this case truly unusual. A study using UWF in 20 patients of pseudoxanthoma elasticum previously reported utility of this imaging technology in documenting other lesions associated with the disease in 72.5% of eyes, which were otherwise undetected with standard fundus photography.^[1]^ These peripheral lesions included peaud', coquille d'oeuf, cracked eggshell, comet lesions, peripheral retinal degenerations, parastreak atrophies, and peripheral hemorrhages. Though the authors documented peripheral hemorrhages, they have not reported peripheral AS in this highly vulnerable cohort.^[1]^ It is said that AS may grow in length, but such findings have not been documented with clinical studies.^[2]^

Spectral domain OCT, enhanced depth imaging OCT, SSOCT, and enface OCT (C-scans) have been previously used to document changes at the level of Bruch's membrane that corroborate with histopathological findings reported earlier.^[5,6,7,8]^ Reported features include hyper-reflection, undulations, and large dehiscence of the Bruch's membrane. A longitudinal study reports presence of undulations on OCT as a sign of pressure points that would lead to future dehiscence of the Bruch's membrane and then possibly CNVM.^[5]^ We used SSOCT to scan the temporal area of the right eye harboring the peripheral AS noted in UWF imaging and findings were similar to what has been reported earlier.^[5,6,7,8]^ Eyes with AS are prone to retinal hemorrhages, even following insignificant ocular contusions.^[1,4]^ Such bleeds may be seen in up to 5% of the cases and may cause vision loss.^[1]^ Dehiscence in the RPE–Bruch's membrane complex are a known feature of AS.^[5]^ As undulations represent a weak area and are prone to rupture,^[1,5]^ a peripheral dehiscence of the RPE-Bruch's membrane complex near an undulation [Figure 3] explains the peripheral location of the retinal hemorrhages seen in this patient far away from the CNVM. This pathology may be very similar to retinal hemorrhages occurring in high myopia following the development of lacquer cracks.^[9]^ UWF red free imaging clearly indicated that the peripheral AS [Figure 5] were extensions of the central anomaly and not a singular occurrence. These areas may also be a nidus for future peripheral CNVMs, though no CNVM was detected with OCT angiography or fluorescein angiography in that area.

Given the idiopathic nature of AS in this patient, their peripheral extension is rather unusual. Our initial workup for systemic associations did not reveal any finding to suggest a vulnerability for the AS to assume a gigantic form. After leading questions and inter-departmental referrals, the only finding on clinical workup was the long-term steroid abuse for cosmetic reasons. Steroids are known to alter serum calcium balance due to multiple mechanisms.^[10]^ There are specific reports citing hypercalcemia following the use of anabolic steroids.^[11,12]^ It has also been theorized that high serum calcium levels may be responsible for AS in patients with Paget's disease.^[2]^ Though current serum calcium levels were normal in this patient, they may have fluctuated earlier, resulting in the aggressive presentation and increased fragility of the otherwise elastic membrane. Further, steroids can also affect wound healing of the damaged chorioretinal tissue and impair ocular response to undulations and breaks of the Bruch's membrane.^[13]^ However this is an isolated case report, we conjecture that long-term abuse of systemic corticosteroids may have perpetuated the progression of streaks to assume the giant extensions seen in our patient. In future, it may be worthwhile to determine if there is any relationship between corticosteroid metabolism in the body and AS.

To conclude, AS can rarely extend to the peripheral areas of the fundus and be a risk factor for not only central but peripheral vision threatening complications. Role of steroids in aggravating disease progression needs to be further elucidated with larger series.

##  Financial Support and Sponsorship

Nil.

##  Conflicts of Interest

There are no conflicts of interest.
